# Educators' perceived barriers and facilitators to implementing a school-based nutrition, physical activity, and civic engagement intervention: a qualitative analysis

**DOI:** 10.3389/fpubh.2025.1616483

**Published:** 2025-06-27

**Authors:** Andrew McNeely, Alexandra MacMillan Uribe, Gabrielli T. De Mello, Andres Herrero-Loza, Mahak Ali, Kaitlyn Nguyen, Yetunde Olawuyi, Chad D. Rethorst, Rebecca A. Seguin-Fowler, Jacob Szeszulski

**Affiliations:** ^1^Institute for Advancing Health through Agriculture, Texas A&M AgriLife Research, Dallas, TX, United States; ^2^Department of Biomedical Sciences, Texas A&M University, College Station, TX, United States; ^3^Department of Economics, Texas A&M University, College Station, TX, United States; ^4^Department of Nutrition, Texas A&M University, College Station, TX, United States; ^5^Institute for Advancing Health through Agriculture, Prairie View A&M University, Prairie View, TX, United States

**Keywords:** implementation science, exercise, healthy eating, school-based intervention, physical activity

## Abstract

**Introduction:**

Strong Teens for Healthy Schools (STHS) is a middle school program that focuses on improving healthy eating habits, physical activity, and engages students in civic engagement projects to promote healthy environments within schools. As a novel approach to school-based health interventions, this program faces numerous intervention implementation challenges.

**Methods:**

To assess potential barriers and facilitators to implementing STHS, interviews with Texas Cooperative Extension staff (*n* = 20) and middle school staff (*n* = 15) were conducted prior to implementing the program to inform program delivery. Participants (89% female, 71% white, mean age 41 ± 9.2 years old) reviewed sections of the curriculum and provided feedback in semi-structured interviews. Open inductive coding, followed by deductive categorization of codes within the Consolidated Framework for Implementation Research framework, grouped responses into themes.

**Results:**

Themes found in the response were: (1) Trainings should emphasize using STHS in a structured setting and highlight the core components of the curriculum to ensure consistent delivery. (2) Variations in capabilities may affect how information is delivered by implementers, as well as how it is received by students. (3) Participants discussed how the physical infrastructure required for STHS could be a challenge in some school contexts. (4) The STHS curriculum received positive feedback for its design, relative advantage compared to other curricula, and evidence-base. (5) Local partners' attitudes and conditions may affect the adoption and implementation of STHS.

**Discussion:**

Overall, participants supported implementation of the STHS but noted several potential challenges that could be addressed prior to implementation.

## Introduction

There is strong evidence for the many benefits of children engaging in physical activity (PA) and developing healthy eating habits, including reduced risks of depression, anxiety, cancer, type 2 diabetes, obesity, and cardiovascular disease (1–3). However, most American youth do not meet PA recommendations, and most school-aged students do not consume enough fruits and vegetables to meet the dietary guidelines (4–6). The discrepancy between PA and healthy eating recommendations and the reality of children's lack of engagement in these behaviors supports the ongoing need for interventions that promote behavior change. Schools are an important setting for promoting health, given their ability to reach many children, regardless of ethnicity, background, or gender. They also provide an opportunity for continuous, intensive interventions over long periods of time (7, 8). Children spend a large proportion of their time in schools—over 6 h per day for nearly 180 days per year (7–9). Additionally, school programs have demonstrated effectiveness in increasing PA (10, 11), positively affecting the consumption of vegetables, fruits, sugar sweetened drinks, and fast food (12–14) and promoting positive youth development among youth (15). However, when developing a novel program for the school setting it is important to understand potential program specific barriers that may promote or impede delivery (e.g., adoption, implementation, sustainment) (16–18).

School-based PA, healthy eating, and positive youth development programs face critical challenges to implementation (19). Several reviews found that some of the most prevalent barriers to program implementation include staff beliefs that conflict with the program's aim (e.g., underlying value of the intervention or program), lack of access to necessary resources, lack of access to necessary resources, supportive leadership, staff buy-in, and understanding of the program's principles and purpose, as well as other specific school characteristics (e.g., school size, high school vs. elementary school) (20, 21). Another study that examined school-based interventions found that ‘inner contextual factors' (i.e., factors within the school) were predominately related to sustainability, including availability of facilities or equipment, continued executive or leadership support, and implementation team cohesion (22). Furthermore, these results are supported by numerous other studies that identify additional barriers to various aspects of implementing school-based interventions, including managing students, inadequate resources, and a lack of time, knowledge, skills, competence, and training (22–24). Conversely, access to resources to support implementation, presence of effective and supportive leadership, access to ongoing training, effective communication about the purpose and outcomes of the program, teacher support, good training, and technical assistance can all facilitate the delivery of school-based programs (20, 22, 25).

Strong Teens for Healthy Schools (STHS) is a middle school program that was adapted from an adult civic engagement and health behavior program, the Change Club, which was created to empower people to take an active role in creating healthier eating and PA environments in their community (26). The STHS curriculum, which uses the theory of planned behavior (27–29), the socioecological model (30–32), and the whole school, whole community, whole child (WSCC) model (33, 34) to address PA, healthy eating habits, and positive youth development, is comprised of 16 one hour sessions, with half devoted to civic engagement skills and half alternating between PA and nutrition topics. One novel component of the curriculum is the student-led project to promote healthy environments within schools. Creating a health-focused change to the built environment through civic engagement is an evidence-based method that has been implemented in both rural and urban communities (35–38). These programs have demonstrated success in facilitating environmental change (39, 40) and have been integrated successfully into evidence-based, multilevel interventions as well (38). STHS adapts its civic engagement curriculum from these prior iterations to target adolescent cardiometabolic health in the school environment. STHS is also aligned with the Texas Essential Knowledge and Skills (TEKS) (41)—curriculum standards set by the state board of education—to help improve adoption of the program in Texas schools.

**Figure 1:**
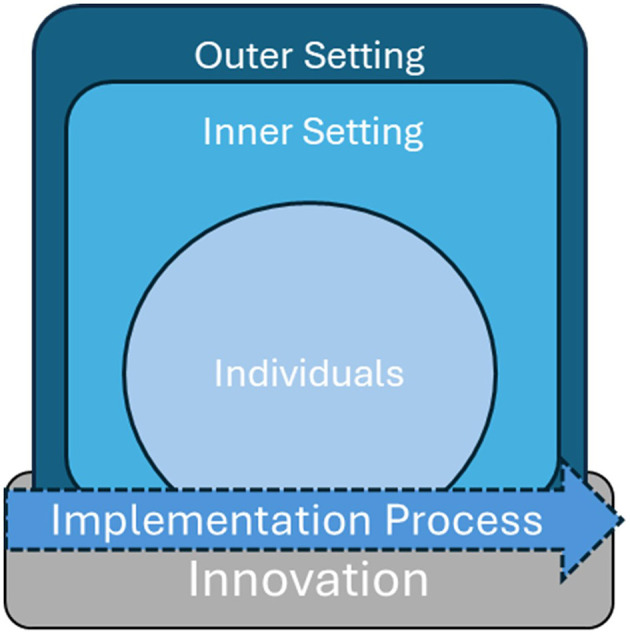
Adapted from the consolidated framework for implementation research (CFIR).

Achieving behavioral changes and promoting health benefits in educational settings requires a comprehensive understanding of factors that may impede or support the delivery of effective programs within school settings (22). Given the novelty of this approach in a school setting, understanding potential barriers and facilitators to its implementation in schools is essential to informing the development of implementation strategies that can enhance the delivery and sustainment of programs over time (22, 42). The Consolidated Framework for Implementation Research (CFIR) (43) is a conceptual model that divides the implementation of a given program into five domains (Figure 1)—the innovation (i.e., the program being implemented); the individuals involved with delivering the innovation, including implementers, leadership, support staff, administrators, recipients, etc.; the inner setting where the innovation is implemented; the outer setting, which includes the surrounding community and context beyond of the inner setting; and the process (i.e., how implementation occurs). Given the complex, multi-level nature of STHS, CFIR provides the most comprehensive understanding of factors that may affect program delivery throughout all phases of implementation (adoption, implementation, and sustainment), whereas many other models are more limited in their focus. Additionally, CFIR has been previously used to help identify and categorize barriers and facilitators to the implementation of other programs in the school setting (44–47) and has successfully helped to generate implementation of other programs and generate implementation strategies that aim to improve program delivery (48).

Overall, school-based interventions to promote health face a number of implementation challenges that can adversely impact the outcomes of such programs (20, 22, 24), but frameworks such as CFIR allow implementers to anticipate and adapt their interventions to overcome barriers (44, 45). However, STHS uses a novel civic engagement approach to school-based health and poses challenges to implementation in the school setting. Therefore, this study investigated the perceived barriers and facilitators to implementing STHS from the perspective of Texas Cooperative Extension staff (i.e., health educators) and middle school staff before it was delivered in schools to inform program delivery.

## Methods

### Overview of setting

The state of Texas school system encompasses more than 9,000 campuses, including 1,475 middle-schools (32). Students in middle-schools comprise about 18% of the total number of enrolled students in Texas schools, and are typically between the ages of 11 and 13 years old. In addition to resources and education about health provided directly by the school, external organizations and agencies can be utilized to implement beneficial programs. In the United States, Cooperative Extension is a nationwide, educational network, operating through land-grant universities in partnership with federal, state, and local governments, that translates research into practical knowledge and action to address public needs. In Texas, this system is collectively referred to as AgriLife Extension, and has a presence in all 254 counties of the state (33). Extension personnel include agents who provide a range of programs and services to the public, and 4H staff that specialize in delivering youth development programs to schools.

### Design

Qualitative interviews were conducted to assess barriers and facilitators to the implementation of STHS. Interview questions were based on CFIR domains. Questions prompted participants for feedback about the innovation (e.g., benefits and drawbacks to the STHS program), its delivery (e.g., usefulness of the materials in aiding instruction), as well as factors that could affect implementation of the STHS program (e.g., perceptions of school leadership support, physical design of the school). Participants were provided with a high-level overview of the STHS program and assigned two (out of 16 total) lessons of STHS to review. Lessons to be reviewed were assigned sequentially (i.e., the first participant received lessons one and two). Participants reviewed their assigned lessons using digital copies of the curriculum and were provided with an evaluation sheet to document their impressions and prompt discussion during the interview. Participants were asked to complete their review within 1 week of assignment but were given additional time if needed. Upon finishing their self-paced review, participants completed a demographic survey assessing gender, race/ethnicity, and years of experience in respective roles (i.e., extension/schools), and scheduled a time to complete a 45-min semi-structured interview with a research team member. This study was approved by the institutional review board at Texas A&M (IRB2022-1159D).

### Recruitment

The research team recruited potential implementers of STHS using a multi-pronged approach beginning with Texas Cooperative Extension staff members (48). Staff members of the Texas AgriLife Extension system (hereafter, Extension staff) that were recruited for this study include county extension agents and 4H staff members, which were identified using AgriLife extension website and word of mouth referral.

Educators were recruited by sending informational emails to contact email addresses on Texas school websites. Convenience sampling was also used to recruit educators directly at school health conference. For both Extension staff and educators, snowball sampling was used, in which participants could share research team contact information with their colleagues who may have been interested in participating in the study. Inclusion criteria for educators were adults (≥18 years old) with any number of years of education experience in a Texas public or private middle school settings (past or present). Participants were provided with an electronic copy of the study information and informed consent information sheet. They provided verbal consent to the researcher prior to beginning the interview. Participants were compensated for their time with a $50 Amazon gift card.

### Data collection

Interviews were conducted online (Microsoft Teams version 25) and recorded by AMU and JS. Audio recordings of the interviews were uploaded to a secure web-based transcription service (NVivo Transcription 2024, Lumivero LLC). Transcript files were cleaned and reviewed for accuracy against the audio recording by a research team member. Final transcripts were then uploaded to qualitative analysis software (ATLAS.ti 25), and copies of the project were shared with coding team members.

### Analysis

All data analysis was led by the first author (AM). Analysis team members (AH-L, MA, KN, GTDM) met with the team leader as well as principal investigators (JS, AMU) regularly to discuss progress and findings. Coding proceeded in three stages. In the first stage, members of the analysis team performed open coding on the transcripts to capture *a priori* ideas and patterns. Second, the same team members applied axial coding to the analysis to sort and refine codes into conceptual content areas (49). The analysis team then engaged in focused, deductive coding, applying the CFIR domain and subdomains to the codes established in the first two rounds of coding. An additional round of line-by-line focused coding for CFIR concepts was performed to ensure all data was accounted for in the analysis. Coded transcript excerpts, categorized by CFIR domain and subdomain, were then reviewed by the analysis team members and the principal investigators, and final analytic themes were developed, reviewed, and approved for each CFIR domain by all team members. Demographic surveys were collected via Qualtrics (Qualtrics, LLC 2024), and exported for analysis in Microsoft Excel (Version 2403).

The use of inductive and deductive methods in the analysis also influenced the method of determining an appropriate cessation of data analysis, also known as saturation in qualitative studies. Following the analysis model, inductive thematic saturation (50), in which no new codes or themes could be derived from the data, was first established. Then, this coding was organized under CFIR domains and subdomains until all data had been accounted for in this framework, achieving a form of *a priori* thematic saturation (50). As expected, inductively derived codes sometimes crossed into multiple CFIR domains and could not be easily organized into these deductive categories. In these cases, the analysis team lead (AM) and the principal investigators (JS and ALMU) discussed the relevant topics and quotations, and made decisions for categorization on a case by case basis.

### Research team characteristics

The research team represents a professionally and individually diverse group. This study was led by faculty members with Texas A&M Institute for Advancing Health through Agriculture (IHA) JS, AMU, CDR, and RAS-F. At the time of interviews, analysis, and writing, AM, GTDM, and YO were postdoctoral researchers with IHA, and AH-L, MA, and KN were undergraduate student researchers at Texas A&M University. All faculty and postdoctoral researchers have had professional experience and a current research agenda in community health, and training and professional experience in qualitative research methods; student researchers were trained in qualitative research methods by AM.

## Results

Extension staff (*n* = 20) and school staff members (*n* = 15) completed qualitative interviews between March of 2023 and March of 2024. Most participants were female, non-Hispanic White, with 6 or more years of experience in their respective roles (Table 1). The average age of participants was 41 years old (standard deviation ± 9.2 years). The interviews lasted an average of 33 min (rounded to nearest minute), with a standard deviation of ±11 min. The shortest interview was 16 min, and the longest interview was 59 min.

*Implementation process theme—Trainings should emphasize using STHS in a structured setting and highlight the core components of the curriculum to ensure consistent delivery*.

**Table 1:** Demographic characteristics of interview participants (*n* = 35).

**Characteristic**	**Extension staff (*n* = 20)**	**School staff (*n* = 15)**	**Total (*n* = 35)**
Age (years), ±SD	42 ± 9.6	40 ± 8.7	41 ± 9.2
**Gender**			***n*** **(%)**
Female	20 (100)	11 (73)	31 (88.6)
Male	0 (0)	4 (27)	4 (11.4)
**Race**			***n*** **(%)**
American Indian or Alaskan Native	1 (5)	0 (0)	1 (2.9)
Black or African American	1 (5)	6 (40)	7 (20)
White	17 (85)	9 (60)	25 (71.4)
Not answered	1 (5)	0 (0)	2 (5.7)
**Hispanic (any)**			***n*** **(%)**
Yes	3 (15)	2 (13)	6 (17.1)
No	17 (85)	13 (87)	29 (82.9)
**Years of experience**			***n*** **(%)**
0–5	6 (30)	3 (20)	9 (25.7)
6–10	8 (40)	5 (33)	13 (37.1)
11+	6 (30)	7 (47)	13 (37.1)
**Role**			***n*** **(%)**
4H staff	7 (35)	–	7 (20)
Extension agent	13 (65)	–	14 (40)
Principal	–	1 (7)	1 (2.9)
Teacher	–	11 (73)	11 (31.4)
Other	–	3 (20)	2 (5.7)

Although STHS is designed to be deliverable either by school staff members or by extension agents, participants emphasized the necessity of training the implementer on the program components, particularly its most critical components, to ensure fidelity. These comments were often made in reference to implementer time constraints and their need for clear expectations about their role (Table 2, Quote 1); implementers balancing multiple demands on their time may inadvertently skip important pieces of the curriculum if they are not emphasized in training. Aside from training, however, participants noted that the curriculum itself was appealing in that it would require very little expert knowledge or preparation time, thereby promoting its consistent use by implementers (Table 2, Quote 2).

**Table 2:** Implementation process theme—trainings should emphasize using STHS in a structured setting and highlight the core components of the curriculum to ensure consistent delivery.

**Quote#**	**Subdomain**	**Representative quote**
1	Assessing needs of innovation deliverers	And sometimes I've seen personally, and with other agents, that important components are omitted because they didn't, they were in a rush to implement and they didn't take the time to read every single part of it. And then they're like “Oh, I was supposed to do that? What week?” And, you know, it's something that could impact the outcome of the program. So the only thing I would recommend is just maybe do, and I know the time is a constraint, right, do you maybe like an initial implementation and then have quarter to year or monthly meetings where you kind of highlight, “okay, you're on this week, this is probably what you need to be doing. Highlight this, this and this.”... But I think it's important, really important when it comes to the training and the implementation of this program or any program for that matter, to really take into consideration that the time you spend with them is probably the most they're going to put into the curriculum until they get used to it. So highlighting things that are extremely important is necessary. -EA2
2	Engaging innovation deliverers	Teacher-wise? I think they'd be like, “Yes, yes, you give me something that I don't have to spend hours upon hours planning, and you know, gathering resources and all of that stuff”. I think they would absolutely be on board. -SS8
3	Tailoring strategies	I think for it to be successful at school, it's going to have to be in the classroom. It's just because sometimes the consistency of after school programs, and they're not required to address the [state education standards], whereas the classroom setting is.−4H1

EA/4H, extension staff participant; SS, school staff participant.

In regard to fitting STHS into the school's programming, participants noted that although after school programs can be beneficial for students, after-school programs lacked consistency in terms of delivery (e.g., student might not attend every day) and educational standards (e.g., after school programs may not align with the same educational standards required in the classroom) that participants believed would be necessary to successfully implement STHS (Table 2, Quote 3). According to the participants, implementing STHS inside a classroom setting, such as an elective or part of physical education or health courses, would be more suitable for consistency of delivery.

*Individuals theme—Variations in capabilities may affect how information is delivered by implementers, as well as how it is received by students*.

For the implementers of the program, interview participants generally affirmed that the STHS curriculum itself was easy to understand and could be adequately delivered by teachers of any experience level. Some participants noted that the educator's level of experience, however, could influence delivery in subtle ways that, while not hindering adequate delivery, might result in varied experiences and engagement. Veteran teachers may be better equipped through training and experience to manage the pace of the sessions to fit allotted times, where novice teachers may struggle with ensuring all content was covered in the same amount of time. Participants also mentioned that in classes with shorter sessions, experienced teachers would know how to add supplemental material to fill out the time, whereas new teachers may be left with gaps in their schedule (Table 3, Quote 1). It was also noted that Extension staff may struggle with the timing more than schoolteachers, as their experiences with program delivery do not always involve middle-school education settings, and thus they may need more guidance on classroom management (Table 3, Quote 2).

**Table 3:** Individuals theme—variations in capabilities may affect how information is delivered by implementers, as well as how it is received by students.

**Quote#**	**Subdomain**	**Representative quote**
1	Innovation deliverers (capabilities)	… So I've been teaching for 10 years, I would find this extremely easy to teach because I have had enough classroom, real world experience to know I need to find other activity activities to supplement this curriculum or add to it… A new teacher coming in would, I think, especially since we have a lot more educators who are alternative certification or they're getting certified through district programs so they don't have college background or any educational like experience coming in, I think they would take it very literally and it would cause classroom management issues because they're underutilizing resources like they would literally do this and then they'd say, “okay, what do I do for the other 20 min of class? I don't know what to do.” -SS9
2	Innovation deliverers (capabilities)	… if you're dealing with extension agents who maybe haven't been teachers before or they're not, I mean they haven't really been in education, especially with this age range, there's probably gonna be needing some more specifics on how to run it through the lesson. -EA4
3	Innovation recipients (needs)	Middle school is rough, and they're facing a whole lot of peer pressure and hormones and families… there may be kids who are not as athletic, but they still need to know about physical activity and good nutrition and how that plays into growing bodies and hormones and all of that as well as they start to grow up and become real people. I think middle age is a really good target age for this curriculum.−4H6
4	Innovation recipients (capabilities)	I just for my experience, with these kids working groups, they are very emotional and they will get in all out fights over just disagreeing about stuff with each other. And they're like best friends and all by the end of the class, they don't hate each other. So just having that kind of like being able to facilitate, mentor the discussions, keep them calm and you know, they are very passionate about food. So they can get heated about stuff and someone says, you know, “I want to get rid of sodas machine”, well that may not be okay with somebody else. They get that argument about it. So that's where it may not be very suitable for this level just because their maturity level, having those conversations in a group can sometimes get a little crazy… But there were good groups like my advanced class. I could do a lot of different things with them that I could not do with my regular kids just because it was it was the maturity level. I hate to say it, but it was. I mean they have conversations with me about topics and stuff where my other classes could not. So, it's an interesting age range. -EA4

EA/4H, extension staff participant; SS, school staff participant.

Participants viewed STHS as a source of critical information and skills for middle-school students. Specifically, the connection between nutrition, PA, and mental health, particularly during this critical developmental age, was a topic from which participants thought many students would benefit (Table 3, Quote 3). Participants also discussed the civic engagement components of STHS as a needed skill for this age group, asserting that such material could be helpful in identity formation for middle school students as they progress through adolescence. On the other hand, aspects of the developmental stage of these years could create an added challenge for engaging middle school students in the curriculum. For example, the wide range of maturity levels among potential students could make it difficult to present the material or expect students to engage appropriately (Table 3, Quote 4).

*Inner setting theme—Participants discussed how the physical infrastructure required for STHS could be a challenge is some school contexts*.

Structural characteristics regarding the layout and facilities of the school were mentioned primarily by school staff participants as being important for the implementation of STHS. In most cases, school staff had no concerns about accommodating the program's physical activities. In some cases, however, implementers who described their school settings as smaller and/or in rural areas, noted that interior space was often multi-purpose, such as gyms combined with cafeterias, which might limit their ability to use those spaces. However, these schools often had abundant outdoors space, which participants noted as a possibility for supplementing the program's space requirements (Table 4, Quote 1).

**Table 4:** Inner setting theme—participants discussed how the physical infrastructure required for STHS could be a challenge is some school contexts.

**Quote#**	**Subdomain**	**Representative quote**
1	Structural characteristics–physical infrastructure	I've struggled with this as the health teacher here. Our gym is also our cafeteria because we're small. So I've wanted to like have the kids make nutrition posters or, you know, and put it up in the cafeteria. But I really can't because it's our gym and we have games… but other than that, we have a great layout where we could do like a we did a run last year, you know, and we have like 25 acres. We're all kind of spread out so we could with portables. -SS2
2	Structural characteristics–physical infrastructure	… if this is done in the physical education class, that class is just in the gym. They don't have desks and workspaces… that particular teacher doesn't have supplies and all of that stuff just right there in their class because their class is the gym… Supplies would have to be brought in and all of that stuff because he doesn't have just a regular classroom. -SS8

EA/4H, extension staff participant; SS, school staff participant.

In addition, the use of classroom lessons and physical activities was seen as an effective, but logistically tricky, combination. For example, although nearly all schools had adequate physical education space, such as a gym, participants discussed how moving between the gym and the classroom during the instructional period may be cumbersome to the implementers. On the other hand, if the program were implemented in a gym by default, additional considerations or planning would be needed to make sure the gym was equipped with the necessary items to facilitate the curriculum's classroom lesson (e.g., desks, screens) that are not typically a part of the physical education space (Table 4, Quote 2).

*Innovation theme—The STHS curriculum received positive feedback for its design, relative advantage compared to other curricula, and evidence-base*.

Participants suggested that STHS would only be feasible to implement if it did not create any new demands for the school staff. For many participants, this was emphasized in the fact that they saw STHS alignment with the TEKS as a benefit of the curriculum. Class time in almost all subjects must be dedicated to meeting the TEKS standards, which are evaluated in statewide standardized testing. Thus, STHS aligned with the requirements and processes already in place in the schools and would not be perceived as taking anything away or adding additional burden to staff (Table 5, Quote 1). It is worth noting that alignment with TEKS was seen as a facilitator to implementation, but several Extension staff members expressed that it was not a guarantee of program adoption by the school due to difficulty implementing any kind of program in schools during state-mandated testing season.

**Table 5:** Innovation theme—The STHS curriculum received positive feedback for its design, relative advantage compared to other curricula, and evidence-base.

**Quote#**	**Subdomain**	**Representative quote**
1	Design	Um, I think it depends on the staff… if it would take the place of something that they already have in their schedules, then I don't think staff would perceive it as like, you know, something extra… -SS1
2	Design	Well, our big push in our school district is going through a blended learning model. And so we're really doing things trying to break away from that “sit and get”... I think this type of program, it's really hit me that its engagement was one of its main components. And I think as long as it doesn't get to where [its] heavily relying on that “sit and get”, students are going to appreciate it more…-SS10
3	Design	And so, I think the structure, at least in the two activities that I reviewed, having some group and some individual activities gives a good balance to where if there is somebody that becomes disengaged with it, there's a way to draw them back in. And so, and that's the main thing is it's got to be inviting. -EA8
4	Relative advantage	… I discussed it with somebody that is teaching health. That was before this came out. And they were always looking for something, programs like that, that will be specific. Sometimes they would just have to search, go online and all that and search for programs, activities. But this one, they would really receive it because one, everything is outlined. You don't have to do a whole lot of online stuff to do it. Yeah, and it's easier to also implement. Sometimes they come up with a program and it's very difficult for you to implement it, you're just going to put it aside and do something else. -SS6
5	Relative advantage	Well, I think a lot of the schools have maybe a nutrition program or a physical fitness program… But in my opinion, I don't know of any program that is this detailed, this diverse. So I think it's one, it shows different avenues and it speaks of healthy eating and exercise and civic engagement. -EA3

EA/4H, extension staff participant; SS, school staff participant.

Participants voiced a nearly unanimous positive reception to the design of the STHS curriculum in terms of its usability (e.g., clear, simple instructions) and thoroughness (e.g., self-contained, all materials provided). In addition, participants thought that the variety of activities and lesson structures would appeal to all types of students and a variety of learning styles. The curriculum was also praised for its minimization of “sit and get” learning, described as students simply sitting and listening to traditional, formal lectures (Table 5, Quote 2). Instead, participants discussed how STHS favored group-based activities, active learning, and physical engagement. The balance of activities was also considered a high point, in that no single learning style or teaching format (e.g., group vs. individual tasks, video vs. reading) stood out above the rest (Table 5, Quote 3).

STHS was also discussed in reference to other programs (or the lack thereof) that addressed similar topics. The nutrition and/or PA information covered by STHS was considered to be specific and actionable by participants, whereas the lessons in comparable programs were often too vague or generalized to be particularly useful to students (Table 5, Quote 4). Furthermore, participants asserted that STHS was overall more comprehensive in its approach, covering a variety of topics relating to nutrition, as well as connecting them to health and social connections in a way that is not typically done within school-based curricula (Table 5, Quote 5).

*Outer setting theme—Local partners' attitudes and conditions may affect the adoption and implementation of STHS*.

In reference to the context in which the school operates, a frequent topic of discussion by Extension staff was the Student Health Advisory Councils (SHACs), which are required by Texas law to be established in each school district and are required to meet at least four times per year. Texas SHACs are comprised of educators and administrators, county extension agents, and local residents. Interview participants cited the SHAC as a logical partner in adopting and delivering the STHS program into schools (Table 6, Quote 1), though some noted that SHACs can be inconsistently administered, understaffed, or overburdened with existing obligations. Comments regarding SHACs—both positive and cautionary—came from Extension staff, consistent with their role as health and education promotion agents within the county who regularly partner with community advisory and steering committees such as SHACs.

**Table 6:** Outer setting theme—local partners' attitudes and conditions may affect the adoption and implementation of STHS.

**Quote#**	**Subdomain**	**Representative quote**
1	Partnerships and connections	Definitely the SHACs would help implement or help implement this program. I know now with all the new regulations that Texas has, SHACs have really been a big help all around when it comes to anything like this. So SHACs, definitely.−4H3
2	Local attitudes	So when you're doing your in-depth plan, if your county says that, you know, heart health is an issue or physical activity is an issue or advocacy is an issue, then this is something that you can implement to help with that issue. But everything that we do on our committees is issue driven. So it's what the community says is important to them.−4H4
3	Local conditions	But there's also so much turnover at the schools that that I think makes it difficult for staff. This year I've got all the principals that I'm working with. I mean, they all change. And so I don't know. You know what? I don't know if we're going to be able to continue working on know programs that we worked on last year. I just don't know what's in store. I didn't even know the name of the principal that was that was hired for one of the schools until just a few days ago. It wasn't announced. So there was no way to even, you know, talk to that person to see… And I know in one of the school districts I work well, two of the school districts I work with, turnover is just unbelievable. You know, for the teachers as well. So I never know who I'm going to be working around and how involved they're going to be and how supportive. -EA11

EA/4H, extension staff participant; SS, school staff participant.

The mandate of the SHAC is to ensure that school district health priorities reflect the priorities and values of community residents. In this regard, participants noted that in their preparation for the academic year, local priorities are given precedence, meaning that programs such as STHS must align with not only the implementer's objectives, but the goals of stakeholders in the broader context as well (Table 6, Quote 2). The implementation challenge for STHS, then, would be in the assumption that SHACs would be a consistent source of support between years. Finally, participants noted that factors outside of their influence or control, such as staff turnover at the district or school administrator level (Table 6, Quote 3), could impede implementation of programs like STHS.

## Discussion

Implementation of evidence-based innovations requires identification of barriers and facilitators to using that program with a specific population in a specific setting. To this end, the CFIR framework was preemptively applied to interviews conducted with Texas middle-school educators and extension staff regarding the STHS program at their respective school(s). The study found that participants provided generally positive comments, with some specific caveats and hesitations, about the potential for implementation of STHS. These findings align with much of the literature on barriers and facilitators to school-based programs; programs are more likely to be successfully implemented when there is support and training available from the program developers^25^, which the participants noted as a positive strategy to promote buy-in from school staff and leadership.

Additionally, aligning programs with state-mandated testing requirements, which several participants mentioned as a strength of STHS, reduces the competition in school priorities, a documented barrier to implementation (25). Based on these findings, it will be important for researchers, and eventually, Extension staff working with schools to adopt STHS to discuss the types of training and resources that will be available to teachers and emphasize the alignment of STHS with TEKS as a way to enhance curricular alignment, and potential academic outcomes. These findings also echo prior implementation evaluation of civic engagement interventions to create health-focus changes to the built environment; important facilitators for success of these types of programs include securing stakeholder support (e.g., partners in the community that can mobilize resources to facilitate change) and being able to negotiate around time constraints (26, 39, 40).

Participants also noted, however, specific circumstances and contexts in four of the five domains that could act as barriers to implementation if not considered. A notable example is the continually shifting priorities of external partners such as SHACs, which as noted are obligated to reflect the values and priorities of local community residents. This is in line with previous research that identifies socio-political contexts and the priorities of the surrounding environment as possible barriers to implementation of school-based programs (22). This is also the space in which Texas Extension staff operate, as many of them serve on their SHAC. By working with extension agents to implement STHS, the research team can identify when schools and communities are interested in implementing health and positive youth development programs, as well as other circumstances where STHS may align with the needs and values of communities (e.g., wanting to offer an after school program). By providing unique insight into the priorities and amenability of their local SHACs and other potential implementation support, extension agents will thus be critical partners in tailoring STHS implementation plan to local contexts.

Another finding was that variability in teachers' or extension agents' experience was a potential barrier to implementing STHS. More specifically, participants stated that newer teachers and extension agents may not be as prepared to manage classroom time and/or students, and these topics were not covered in the curriculum materials. These results corroborate previous studies that also identified managing time and students as a challenge during the implementation of behavioral programs in schools (14, 15, 20). Some methods of addressing this concern may be to provide optional additional training, ancillary support materials, or additional implementation support from an experienced educator to help less experienced implementers with the program. Providing training for the STHS program was perceived as positive implementation support and is widely supported as a facilitator to successful school-based programs (22–24), both in the United States as well as international contexts (51, 52). By providing additional time management and classroom management strategies, the research team may be able to provide a more comprehensive and tailored training regimen that addresses potential barriers. In addition, training models that address diverse experience levels could be proposed.

The application of CFIR to these interviews yielded useful information to contextualize the program and provide potential ways to improve the implementation of STHS at different levels of influence, which can lead to designing programs that are more practical, scalable, and impactful for the school context. Although the study revolved around one specific evidence-based program (i.e., STHS), the findings may be applied to a wider array of school-based programs that focus on PA, healthy eating, and/or positive youth development. For example, identified challenges that result in variations in implementation fidelity of after-school programs or inconsistency of community boards, such as SHACs, are likely to be issues that are encountered by many researchers and practitioners delivering programs in the school setting. Additionally, the need for training to implement programs, sensitivity to limited instructional time, and accommodation of varying teacher experience levels are also factors found here that are not unique to STHS or the Texas public education system. However, future research is needed to better understand and test the relationships between the barriers identified here and various implementation outcomes (e.g., adoption, fidelity, sustainability).

## Limitations

Participants in this study reviewed only two sessions of the full STHS curriculum to provide feedback, and provided with a verbal overview of the entire program. Their perceptions of the program's usefulness, barriers, and facilitators of implementation are limited in this way. However, given the consistency in the comments across interviews that formed, and the themes discussed here, the authors are confident that the findings reflect the overall STHS curriculum and its potential for implementation. Interviews did not consider the perspectives of all people involved with STHS's delivery (e.g., students, parents, school health advisory council members). Although these individuals' perspectives are important, they may not apply to all aspects of the implementation process (e.g., use of the implementation guide, classroom management), and as a result, they are not included here. Future studies providing a more comprehensive perspective of all stakeholders are needed.

## Strengths

Despite not including all pertinent stakeholder groups, as mentioned above, interview participants presented a useful sampling of potential innovation deliverers, facilitators, and support personnel that gave comprehensive feedback on all aspects of the CFIR framework. Another strength was the use of inductive coding, followed by deductive CFIR coding, which allowed the research team to find emergent barriers and facilitators that may not have been captured by an exclusively deductive coding process, which may be overly constrained by *a priori* categories and ideas. Finally, having participants identify potential barriers before STHS implementation and evaluation allowed the team to pre-emptively change the curriculum and the implementation strategies (e.g., developing additional classroom management training materials) before evaluating it in a randomized controlled trial.

## Conclusion

According to Extension staff and school staff members, STHS provides a comprehensive and accessible intervention for improving middle-schooler nutrition and PA knowledge. Many aspects of the program address existing needs and are tailored to overcome implementation barriers for school-based interventions, as recognized by participants who felt the useability of the curriculum and the alignment with teaching standards were advantageous. Further refinement of the implementation process of STHS could be accomplished through additional strategizing with local community advisory boards (e.g., SHACs) and targeted training that is more responsive to educators' needs.

## Data Availability

Data are available from Jacob Szeszulski (Jacob.Szeszulski@agnet.tamu.edu) upon completion of a satisfactory data sharing agreement.
